# Optimization of Nanosecond Laser Polishing Process for TC4-B_4_C Composite Material and Study on Surface Quality

**DOI:** 10.3390/ma19153266

**Published:** 2026-08-02

**Authors:** Jiahang Zhang, Yatao Yang, Lin Zhu, Wenxing Xu, Zhuang Liu

**Affiliations:** 1 School of Pharmacy, Harbin University of Commerce, Harbin 150028, China; zjh1998222@163.com; 2 School of Light Industry, Harbin University of Commerce, Harbin 150028, China; yangyatao23@163.com (Y.Y.); xuwenxing2002@163.com (W.X.); 101787@hrbcu.edu.cn (Z.L.)

**Keywords:** TC4-B_4_C composite material, nanosecond laser polishing, surface roughness, orthogonal experiment, surface quality

## Abstract

To address the problem of difficult surface quality control after introducing the hard reinforcing phase into the TC4-B_4_C composite material, the nanosecond laser polishing technique was employed to treat the surface of the powder-metallurgy-prepared TC4-B_4_C composite material. The influence laws of laser power, scanning speed, and scanning interval on surface morphology, roughness, microstructure and surface properties were studied. With the three-dimensional surface arithmetic mean height Sa as the core evaluation index, SEM, EDS, microhardness, and XRD were combined to analyze the polishing effect and the evolution of the surface structure. The results showed that the laser power mainly affected the heat input intensity, the scanning speed mainly regulated the degree of heat accumulation, and the scanning interval determined the overlapping state of the scanning path. The three factors jointly influenced the surface remelting and recrystallization behavior. The orthogonal test results indicated that the influence order of each factor on surface roughness was laser power, scanning speed, and scanning interval; the optimal process parameters were a laser power of 30 W, a scanning speed of 750 mm/s, and a scanning interval of 0.025 mm. Under these parameters, the Sa of the sample surface decreased to 2.1 μm, which was 64.9% lower than that of the unpolished sample. Further characterization revealed that the surface roughness accumulation, pores, and irregular undulations were significantly reduced after nanosecond laser polishing, forming a relatively continuous and dense remelting layer. The microhardness increased from 704 HV to 829.3 HV. XRD analysis indicated that the surface layer after polishing was mainly composed of TiO_2_, TiC, and Ti-based related phases, demonstrating that the nanosecond laser polishing mainly improved the surface quality of the composite material through surface remelting, structure reconstruction, and adjustment of the oxidation state. The research results can provide process references for the surface quality processing of TC4-B_4_C and other difficult-to-machine titanium-based composite materials.

## 1. Introduction

The TC4 titanium alloy is widely used in aerospace, shipbuilding, and precision equipment due to its high specific strength, good corrosion resistance, and high-temperature performance [1]. However, under complex service conditions such as high friction and high load, the traditional TC4 titanium alloy still has problems concerning insufficient hardness and limited wear resistance [2]. By introducing ceramic reinforcing phases such as B_4_C to prepare titanium-based composites, the hardness, wear resistance, and load-bearing capacity of the material can be effectively improved, which is an important way to enhance the comprehensive performance of titanium alloys [3].

However, the introduction of the hard reinforcing phase also significantly increases the processing difficulty of the material [4,5]. The TC4-B_4_C composite material belongs to a typical multiphase heterogeneous system. There are differences in hardness, thermal properties, and interface bonding state between the reinforcing phase and the matrix. During traditional finishing processes for titanium matrix composites, such as mechanical grinding or electrical discharge machining (EDM), the extreme hardness difference between the ceramic reinforcing phase and the titanium matrix typically leads to severe tool wear, uneven material removal, pore exposure, and subsurface mechanical damage. Therefore, improving the surface quality of this type of multiphase composite without introducing secondary damage is a key engineering challenge. Compared to these traditional contact-based or erosive methods, nanosecond laser polishing serves as a highly promising, tool-wear-free alternative. By utilizing transient thermal energy to selectively melt the surface layer, it can effectively seal initial pores rather than exposing them, presenting a unique advantage for finishing heterogeneous composite materials.

Nanosecond laser polishing is a non-contact surface-finishing technology that achieves surface refinement by utilizing laser energy to cause transient melting, create a flow of the molten material, and facilitate rapid solidification on the material’s surface, thereby reducing surface peak–valley differences and improving surface morphology [6]. Compared with traditional mechanical polishing, nanosecond laser polishing has the advantages of high processing efficiency, no mechanical contact, and strong adaptability to complex surfaces. However, the quality of nanosecond laser polishing is highly sensitive to process parameters. When the heat input is insufficient, it is difficult to form a continuous molten pool on the surface, resulting in a limited polishing effect; when the heat input is too high, over-melting, ablation, and repeated solidification may occur [7]. Therefore, it is necessary to clarify the influence laws of key process parameters on the surface quality of TC4-B_4_C composite materials and further reveal the evolution mechanism of the surface structure and properties.

In recent years, scholars both at home and abroad have conducted extensive research on the design of reinforcing phases, preparation processes, and performance improvement of TC4 titanium-based composites. Zhou Haixiong et al. [8] used the discharge plasma sintering process to prepare graphene/TC4 composites, finding that the addition of graphene could refine the matrix grains and enhance the compressive properties of the composite by reacting to form TiC particles. Zhang Weichen et al. [9] used selective laser melting technology to prepare in situ self-generated TiB + La_2_O_3_/TC4 titanium-based composites, and the results showed that the introduction of the reinforcing phase could improve the microhardness and high-temperature compressive strength of the material. Regarding the B_4_C reinforcing system, Li Shaolong et al. [10] used TC4 and B_4_C powders as raw materials and applied discharge plasma sintering and hot extrusion processes to prepare TiB- and TiC-reinforced TC4-based composites, suggesting that as the B_4_C content increases, the material grains are refined, and the room temperature and high-temperature strength are both improved; Zhang Wenshuo et al. [11] used direct laser deposition technology to prepare B_4_C/TC4 titanium-based composites and found that the ratio of micron and nano B_4_C particles would affect the crystallinity of TiC and the microhardness of the interface. These studies show that ceramic reinforcing phases such as B_4_C can effectively improve the microstructure and mechanical properties of TC4-based composites, but the introduction of hard reinforcing phases also further increases the difficulty of surface finishing the material.

In the field of laser polishing, studies have shown that this technique can effectively reduce the surface roughness of materials and improve surface integrity through surface remelting, melt flow, and rapid solidification. Bhaduri et al. [12] performed laser polishing on 3D-printed stainless steel parts and found that the maximum reduction in surface roughness exceeded 94%, and they pointed out that the initial surface roughness would affect the polishing effect. Yao Jianhua et al. [13] used a nanosecond laser to polish the high-roughness surface of a piece of 316 L stainless steel after wire cutting, reducing the Ra from 3.79 μm to 0.95 μm. They believed that the melting and leveling of small protrusions on the surface was an important factor behind the reduction in roughness. Liu et al. [14] performed nanosecond laser polishing on 5A06 aluminum alloy, and the results showed that the surface roughness decreased from 1.04 μm to 0.53 μm. At the same time, the changes in the microstructure of the remelting zone affected the surface hardness. Chen et al. [15] performed nanosecond pulse laser polishing on Al/PLA additive manufacturing parts, reducing the surface roughness from 5.64 μm to 0.32 μm. This indicates that nanosecond laser polishing has good applicability for the surface finishing of composite materials. For titanium alloy materials, Ma et al. [16] and Obeidi et al. [17] respectively conducted laser polishing research on additive manufactured Ti alloys and Ti-6Al-4V parts prepared by LPBF, both proving that laser remelting can effectively reduce surface roughness and improve surface conditions. The general mechanism of laser polishing has been summarized as “surface micro-melting—melt filling—rapid solidification”, and parameters such as laser power, scanning speed, and spot size will significantly affect the state of the remelting layer and the final surface quality.

However, most of the existing research has focused on stainless steel, aluminum alloys, additive manufacturing titanium alloys, and polymer-based composite materials. The research on nanosecond laser polishing of TC4-B_4_C multiphase composite materials prepared by powder metallurgy is still relatively insufficient. Compared with homogeneous metal materials, the matrix, ceramic reinforcing phase, and sintering aid-related phases in TC4-B_4_C composite materials have differences in hardness, thermal conductivity, melting behavior, and interface bonding state. Under laser action, problems such as uneven local thermal response, insufficient continuity of the remelting layer, and changes in the oxidation state are more likely to occur. Therefore, it is necessary to further study the influence laws of nanosecond laser polishing parameters on the surface morphology, roughness, microstructure, and surface properties of TC4-B_4_C composite materials. Existing studies have also shown [18] that for Ti-6Al-4V and other titanium alloy additive manufacturing components, surface roughness and pore characteristics will affect fatigue life. This further indicates that surface quality control is of great significance for the service reliability of titanium-based materials.

Based on this, this study took the “90 wt% TC4 + 5 wt% B_4_C + 5 wt% SiO_2_” composite material obtained through the powder metallurgy method and selected for previous screening as the research object. The surface of this material was subjected to surface finishing treatment using the nanosecond laser polishing method. Through single-factor experiments, the influence laws of laser power, scanning speed, and scanning interval on surface morphology and roughness were analyzed, and orthogonal experiments were further used to optimize the process parameters. Combined with SEM, EDS, microhardness, and XRD, the surface microstructure, elemental characteristics, hardness changes, and phase composition under the optimal parameters were characterized. The intrinsic relationship between surface recrystallization, microstructure reconstruction, and performance improvement during the nanosecond laser polishing process was clarified, providing a reference for the surface quality control and laser finishing process optimization of TC4-B_4_C composite materials.

The novelty of this work lies in three main aspects: (1) This is one of few systematic studies exploring the nanosecond laser polishing process specifically for multiphase powder-metallurgy-derived TC4-B_4_C composites. (2) The coupled effects of laser power, scanning speed, and scanning interval on surface quality were quantitatively clarified, defining a practical process window. (3) The intrinsic relationship between localized remelting behavior, defect healing, and hardness enhancement was established, providing theoretical guidance for the laser finishing of titanium matrix composites.

## 2. Experimental Materials and Methods

### 2.1. Experimental Materials and Laser Polishing Equipment

The experimental materials used in this study were TC4-B_4_C composite materials that were previously prepared and selected through the powder metallurgy process. The composition ratio of these materials was 90 wt% TC4 + 5 wt% B_4_C + 5 wt% SiO_2_. Previous research indicates that when the B_4_C content is 5 wt%, the composite material exhibits superior comprehensive performance in terms of hardness and density. Therefore, the sample with this composition ratio was chosen as the object for subsequent nanosecond laser polishing experiments. The sample was a cylindrical block with a diameter of approximately 10 mm and a height of approximately 5 mm. Due to the presence of certain oxide layers, pores, and uneven undulations on the surface of the powder-metallurgy-sintered samples, to reduce the influence of the initial surface state differences on the laser polishing results, the surface of the sample needs to be uniformly pre-treated before the experiment.

During the pretreatment process, the sample surface to be processed was first wet-ground using 500# and 800# sandpapers to remove the surface oxide layer, loose particles, and shallow local defects, thereby obtaining a relatively flat reference surface with consistent scratch orientation. Water was continuously supplied during grinding for cooling, and after each sanding step, the sample surface was rinsed thoroughly under running water to prevent residual coarse particles from affecting subsequent treatments. Subsequently, the sample was ultrasonically cleaned in anhydrous ethanol for 10 min to remove any remaining abrasive debris or fine particles from the surface and pores. After cleaning, the sample was dried at 60 °C for 1 h and then sealed for storage. Following this pretreatment, large-scale pits, particle agglomerations, and irregular surface undulations on the original sample surface were significantly reduced, helping ensure consistency in the initial surface conditions across different experimental groups.

The nanosecond laser polishing experiment was conducted using the Diaotu B3 fiber laser processing system (Diaotu, China). This system mainly consists of a computer control unit, a fiber laser, a galvanometer scanning system, a mirror assembly, a focusing field lens, and a processing platform. During processing, the laser beam is collimated and then directed into the two-dimensional galvanometer system, where it is deflected to follow a preset scanning trajectory, ultimately focusing on the sample surface via the focusing field lens. The experimental parameters—such as laser power, scanning speed, scanning path, and scan spacing—are set using the accompanying Diaotu Industrial control software (Diaotu B3) to achieve controlled polishing of the sample surface. The main laser parameters are shown in Table 1.

### 2.2. Experimental Design for Nanosecond Laser Polishing

During the nanosecond laser polishing process, the surface of the composite material undergoes rapid heating, local melting, melt flow, and rapid solidification under the action of the laser. The final polishing effect is jointly influenced by the intensity of heat input, the duration of heat action, and the overlapping state of the scanning paths. To systematically analyze the influence laws of key process parameters on the surface quality of TC4-B_4_C composite materials, this paper adopts the experimental approach of “single-factor experiment screening-orthogonal experiment optimization” to conduct nanosecond laser polishing experiments. Firstly, through single-factor experiments, the influence laws of laser power, scanning speed, and scanning spacing on the surface morphology and roughness are investigated separately. On this basis, the range of factor levels for the orthogonal experiment is determined, and the optimal process parameter combination is further obtained.

Based on the energy input characteristics during the nanosecond laser polishing process, this paper selects laser power, scanning speed, and scanning interval as the main factors for investigation. Among them, laser power directly determines the amount of energy input to the material surface per unit time, mainly affecting the degree of surface melting; scanning speed determines the duration of the laser beam’s action in a unit area, influencing the level of heat accumulation and the stability of the molten pool; scanning interval determines the degree of overlap between adjacent scanning paths, affecting the continuity of the surface recrystallization layer and the uniformity of energy distribution. To reduce the influence of non-investigated variables on the experimental results, during the experiment, parameters such as laser repetition frequency, pulse width, scanning times, incident mode, and processing area were kept unchanged. The specific fixed parameters are shown in Table 2.

In terms of the scanning path, this paper adopts an arch-shaped scanning method to cover the surface of the sample in a grid-like manner. Compared with unidirectional linear scanning, arch-shaped scanning enables adjacent scanning lines to continuously cover the sample surface in an alternating reverse manner, thereby reducing the idle travel and improving the processing efficiency; at the same time, this scanning method can weaken the melt directional flow and local heat concentration caused by single-directional scanning, which is conducive to improving the uniformity of energy deposition and the continuity of the remelting layer. In the main paper, linear scanning and arch-shaped scanning were compared, and it was pointed out that arch-shaped scanning can reduce the risk of local accumulation or depression and is more likely to achieve a uniform heat field distribution and stable processing repeatability. Therefore, this paper selects arch-shaped scanning as the unified scanning path.

To clarify the influence of each individual process parameter on the polishing effect, a single-factor experiment was conducted using the control variable method. During the experiment, when studying a certain parameter, the other two parameters remained unchanged. In the laser power experiment, the scanning speed was fixed at 500 mm/s, the scanning interval was fixed at 0.02 mm, and the laser power was set at 10, 20, 30, 40, and 50 W; in the scanning speed experiment, the laser power was fixed at 40 W, the scanning interval was fixed at 0.02 mm, and the scanning speed was set at 400, 500, 600, 700, and 800 mm/s; in the scanning interval experiment, the laser power was fixed at 40 W, the scanning speed was fixed at 700 mm/s, and the scanning interval was set at 0.01, 0.02, 0.03, 0.04, and 0.05 mm. The parameters of the single-factor experiment are shown in Table 3.

Single-factor experiments can reflect the independent influence of each parameter on the polishing quality. However, the nanosecond laser polishing process is not determined solely by the action of a single parameter; rather, it is jointly determined by the laser power, scanning speed, and scanning interval, which collectively affect the heat input to the material’s surface layer, the state of the molten pool, and the interconnection relationship of the scanning paths. Therefore, it is difficult to determine the optimal process combination under the condition of multi-parameter coupling based solely on single-factor experiments. Based on the optimal parameter range obtained from single-factor experiments, further L9(33) orthogonal experiments were conducted to optimize the nanosecond laser polishing process. The orthogonal experiments still used laser power, scanning speed, and scanning interval as the evaluation factors, with each factor set at 3 levels. The specific factor levels are shown in Table 4. The specific experimental combinations and Sa test results are presented in Section 3.2.

To ensure the comparability of the results from single-factor experiments and orthogonal experiments, this paper adopts a unified surface quality evaluation method. The arithmetic mean height Sa of the three-dimensional surface is used as the core evaluation index to characterize the overall roughness change of the polished area; at the same time, in combination with surface morphology and subsequent microscopic structure characterization, a comprehensive judgment is made on the uniformity of the polished area, the continuity of the remelting layer, and the distribution of typical defects. When analyzing the results of orthogonal experiments, Sa is taken as the response value, and through the range analysis, the influence degrees of laser power, scanning speed, and scanning interval on the polishing quality are compared, and the better parameter combinations are further selected.

### 2.3. Characterization and Evaluation Methods

To comprehensively evaluate the influence of nanosecond laser polishing on the surface quality and layer properties of TC4-B_4_C composite materials, this paper characterizes the surface morphology, three-dimensional roughness, microstructure, elemental characteristics, microhardness, and surface phase composition. The macroscopic surface morphology was observed using the HG-1501T digital microscope (Zhongte Precision Instrument, Guangdong, China) to determine the overall uniformity of the polishing area, scanning marks, local ablation, and distribution of surface defects; the three-dimensional morphology and roughness were tested using the DSX1000 optical digital super-depth microscope (Olympus, Tokyo, Japan) to quantitatively analyze the changes in surface peak and valley fluctuations before and after laser polishing.

The roughness evaluation is based on the three-dimensional arithmetic mean height Sa as the core indicator, while the root mean square height Sq and the maximum height Sz are selected as auxiliary indicators. Among them, Sa is used to represent the overall level of the surface height deviation from the average plane in the measurement area, which can reflect the overall polishing effect; Sq is more sensitive to local peak–valley fluctuations and can assist in judging the degree of surface undulation; Sz represents the maximum peak–valley height difference and can be used to identify local extreme defects. Therefore, in this paper, Sa is used as the main evaluation indicator for single-factor experiments and orthogonal experiments and is combined with Sq, Sz, and three-dimensional morphology to make a comprehensive judgment on the surface quality. It should be noted that to ensure the statistical reliability of the evaluation, the surface roughness (Sa) for each sample was measured at three randomly selected locations within the polished area, and the average value was recorded and reported. Furthermore, unlike two-dimensional line roughness, the three-dimensional arithmetic mean height Sa inherently represents a statistical average of the surface peak–valley deviations over the entire optical measurement area. The measurement uncertainty primarily stems from the inherent local surface heterogeneity of the multiphase powder-metallurgy-derived composite. However, the consistent macroscopic trends observed across different processing parameters demonstrate the reliability of the presented results.

The surface microstructure and elemental characteristics were analyzed using the Sigma 300 field emission scanning electron microscope (Carl Zeiss, Oberkochen, Germany) and its accompanying EDS energy spectrometer. The focus was on observing the changes in surface pores, rough accumulation, irregular undulations, and remelting layer morphology before and after polishing, as well as analyzing the distribution of main elements on the surface layer and local oxidation characteristics. For cross-sectional observation, the sample treated with the optimal parameters was cut perpendicular to the polished surface, mounted, ground, and polished before SEM examination. The thickness of the laser-modified remelted layer was measured at three different positions from the cross-sectional SEM image, and the result was expressed as mean ± standard deviation. The microhardness was measured using the THVS-1000 Vickers hardness tester (Beijing Time Sihe Technology Co., Ltd., China), with a load of 1 kgf and a holding time of 10 s. Six test points were selected in the polishing area for each sample, and the average value was taken. The surface phase was analyzed using an X-ray diffractometer (ADVANCE, Bruker, Germany), with a scanning range of 20° to 90°, to compare the composition and relative peak strength changes of the surface layer before and after polishing.

In terms of data analysis, single-factor experiments were used to clarify the independent influence laws of laser power, scanning speed, and scanning interval on surface quality; orthogonal experiments used Sa as the response value and, through range analysis, determined the degree of influence of each factor on the polishing effect and the optimal parameter combination. The final determination of process parameters does not solely rely on roughness values but takes into account the reduction amplitude of Sa, surface uniformity, defect control, continuity of the recrystallization layer, microhardness, and phase change in order to ensure the completeness and reliability of the evaluation results.

## 3. Results and Discussion

### 3.1. The Influence of Process Parameters on Surface Topography and Roughness

(1) The influence of laser power

**Figure 1:**
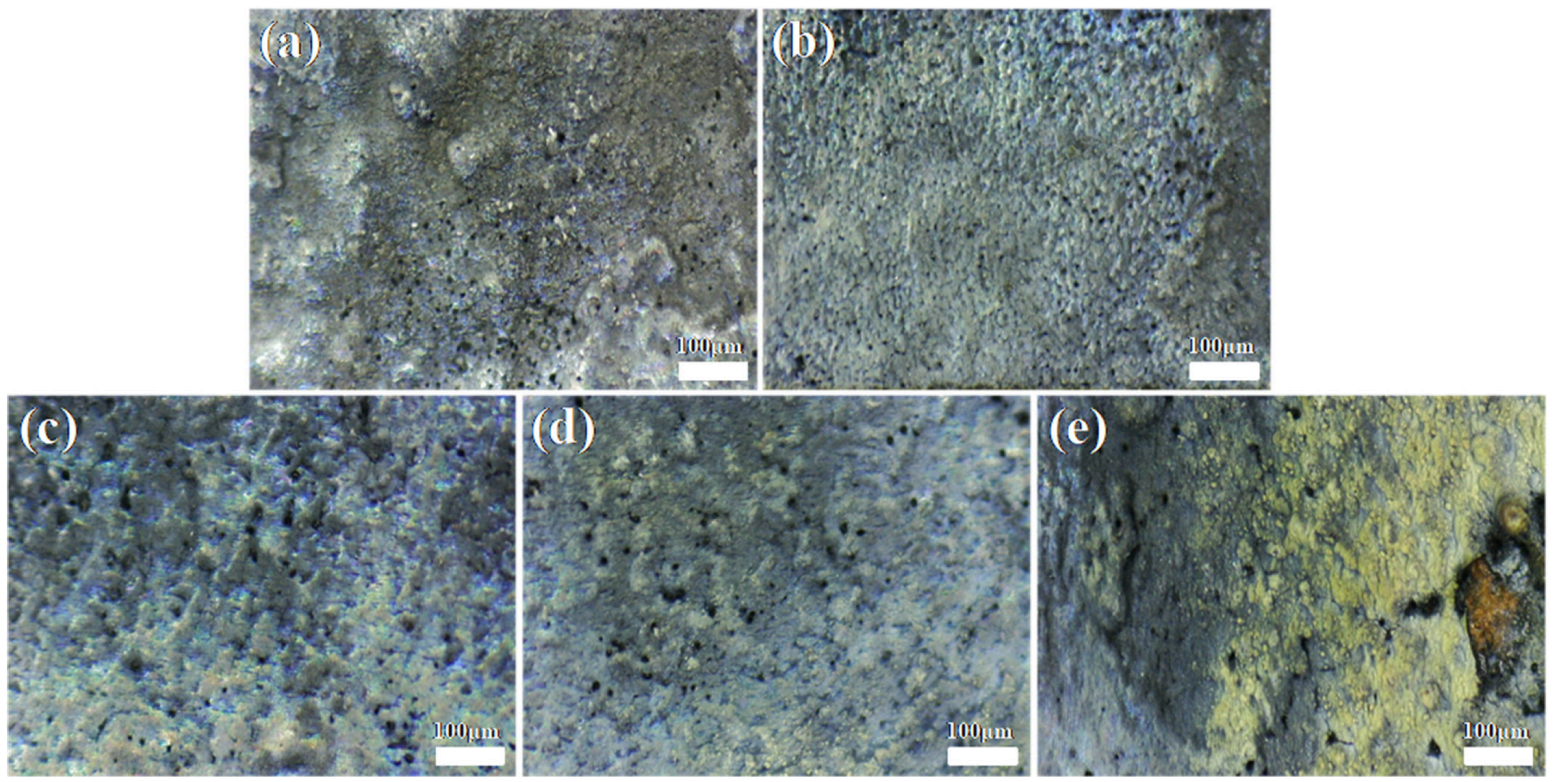
Changes in the surface morphology of the composite material under different laser powers (scale bar = 100 μm): (**a**) 10 W; (**b**) 20 W; (**c**) 30 W; (**d**) 40 W; (**e**) 50 W.

**Figure 2:**
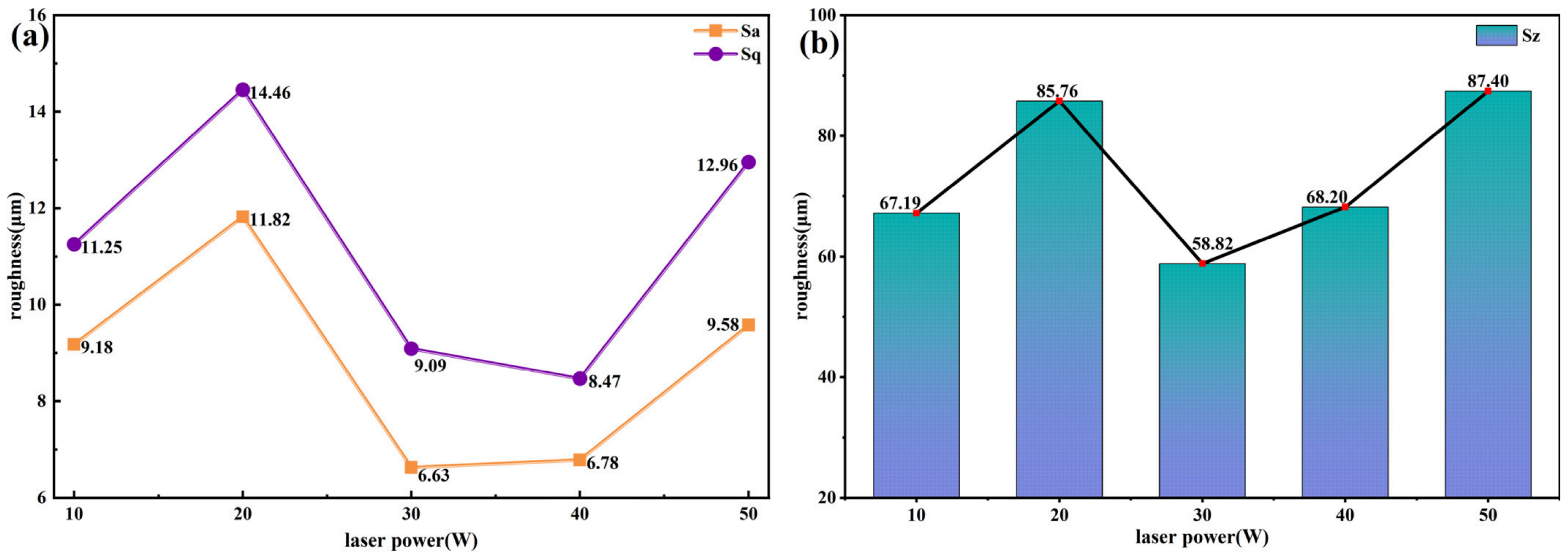
Changes in surface roughness of the composite material under different laser powers: (**a**) roughness Sa, Sq; (**b**) maximum height Sz.

Figure 1 shows the changes in the surface morphology of the composite material under different laser powers, and Figure 2 shows the changes in the surface roughness of the composite material under different laser powers.

As shown in Figure 1, the laser power has a significant impact on the surface morphology of the TC4-B_4_C composite material. It should be noted that for these five different laser power levels, the scanning speed and scanning interval (hatch spacing) were maintained constant at 500 mm/s and 0.02 mm, respectively. When the power is 10 W, there are still many pores, particle boundaries, and irregular undulations on the sample surface, demonstrating that the heat input is insufficient at low power levels, making it difficult to form a continuous remelting layer on the surface and limiting the polishing effect. After the power is increased to 20 W, local melting and recrystallization features appear on the surface, but the overall distribution is still uneven, with melting accumulation and shallow undulations in some areas, suggesting that the continuity of the molten pool and the flowability of the molten material are still insufficient at this time.

When the power is increased to 30–40 W, the surface morphology significantly improves, with fewer pores and irregular protrusions, and enhanced surface continuity. This is because the appropriate heat input causes the surface peaks to melt first, and the molten material flows towards the low-lying areas under the action of surface tension, thereby reducing the peak–valley difference and achieving leveling. Among them, the surface undulation decreases significantly under the 30 W condition, and the remelting is more sufficient and the morphology distribution more uniform under the 40 W condition. When the power continues to increase to 50 W, surface ablation and local roughening occur, demonstrating that excessive heat input will cause over-melting, vaporization splashing, and unstable recrystallization, which instead reduces the surface quality.

As shown in Figure 2, the roughness variation is basically consistent with the morphological evolution. As the power increased from 10 W to 50 W, Sa, Sq, and Sz generally showed a trend of first increasing, then decreasing, and then increasing again. When the power increased from 10 W to 20 W, Sa increased from 9.18 μm to 11.82 μm, and Sq increased from 11.25 μm to 14.46 μm, demonstrating that local non-uniform melting would exacerbate surface undulation. When the power was 30 W, Sa dropped to the lowest value of 6.63 μm, and Sz dropped to 58.82 μm, suggesting that the surface peak–valley difference was effectively weakened; at 40 W, Sq dropped to the lowest value of 8.47 μm, which implies that the surface undulation distribution became more uniform. When the power was increased to 50 W, Sa, Sq, and Sz rose to 9.58 μm, 12.96 μm, and 87.40 μm, respectively, further demonstrating that excessive power leads to deterioration of surface quality.

In conclusion, the laser power does not simply enhance the polishing effect monotonically; rather, there is a reasonable range of heat input. Insufficient remelting occurs when the power is too low, and excessive power can lead to ablation and fluctuations in recrystallization. Considering the roughness and surface morphology, the polishing effect is better within the range of 30–40 W; taking into account that the surface remelting is more sufficient and the surface morphology is more uniform at 40 W, the subsequent experiments on scanning speed and scanning interval selected 40 W as the reference power.

(2) Influence of scanning speed

**Figure 3:**
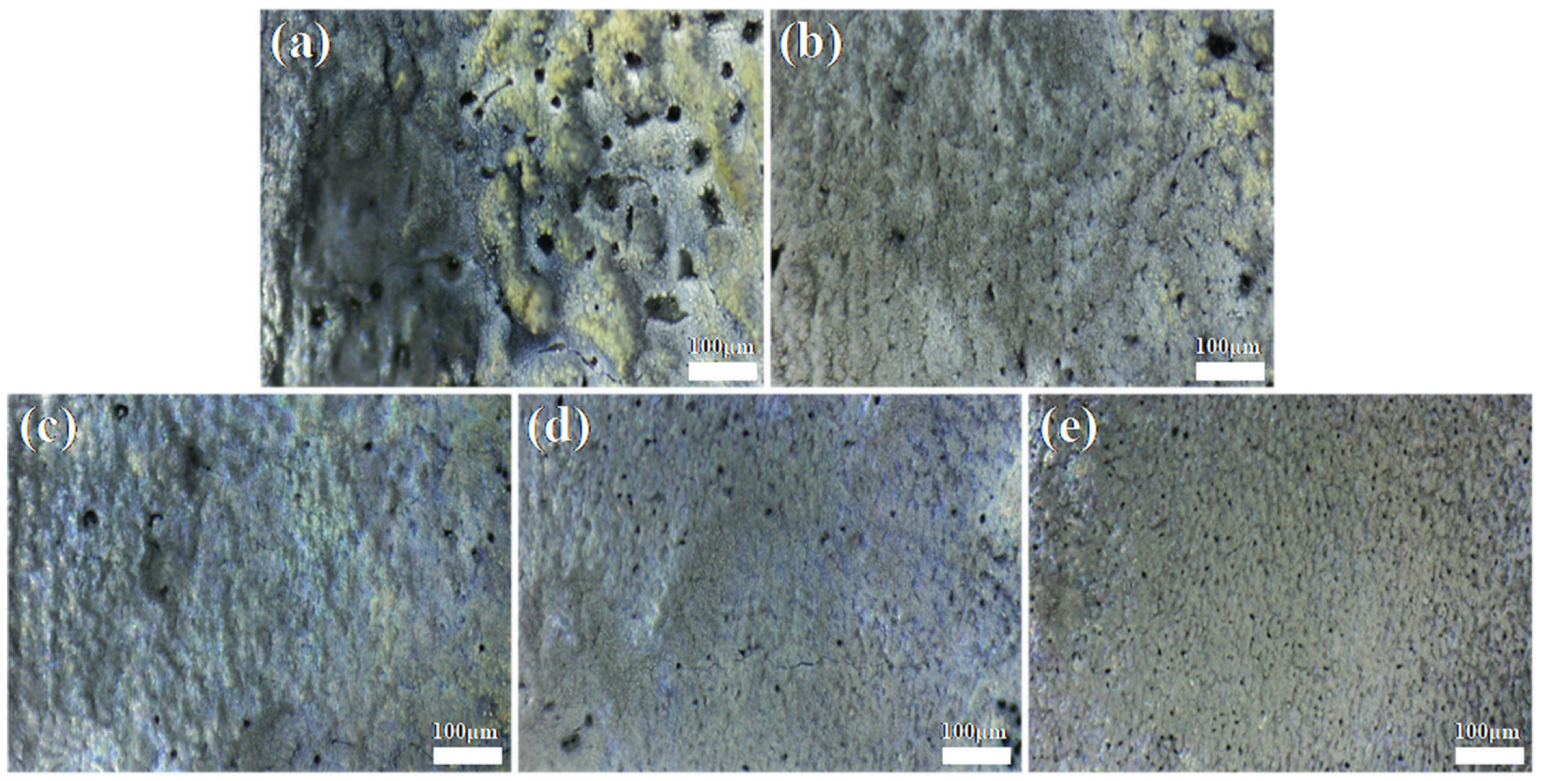
Morphological changes of the composite material surface under different scanning speeds (scale bar = 100 μm): (**a**) 400 mm/s; (**b**) 500 mm/s; (**c**) 600 mm/s; (**d**) 700 mm/s; (**e**) 800 mm/s.

**Figure 4:**
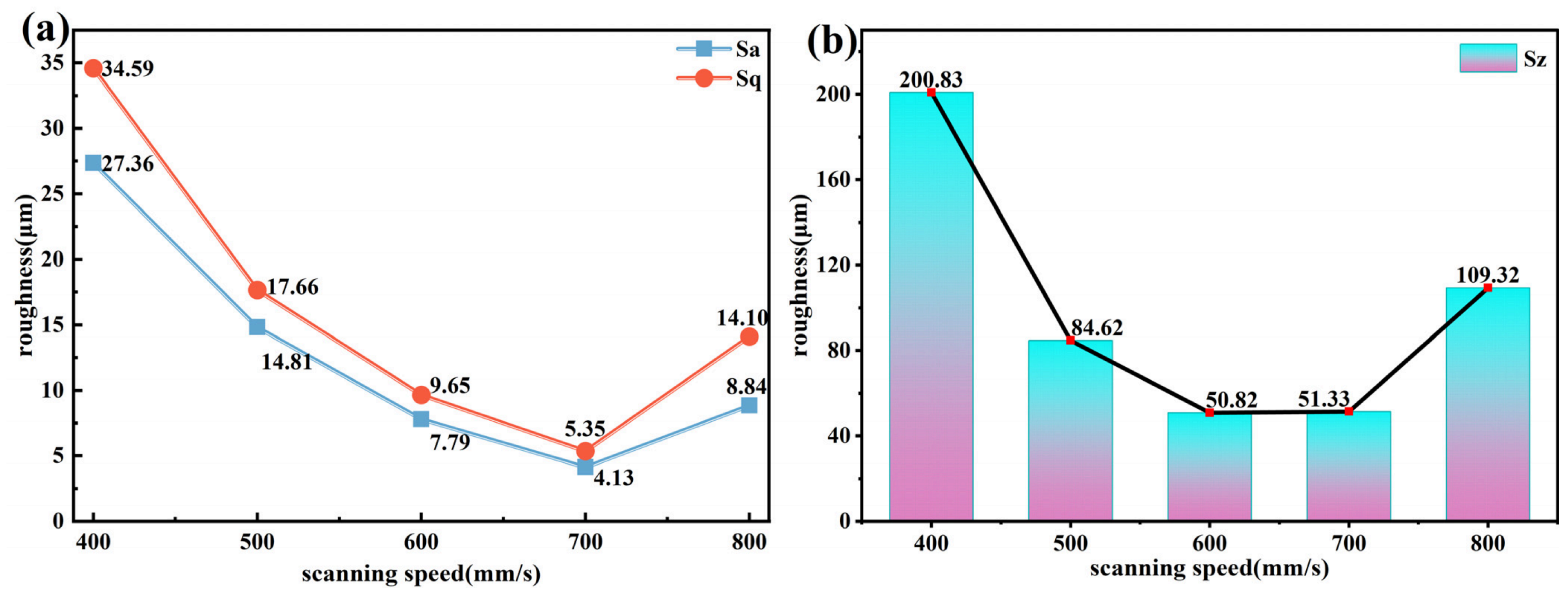
Changes in surface roughness of composite materials under different scanning speeds: (**a**) roughness Sa, Sq; (**b**) maximum height Sz.

Figure 3 shows the changes in the surface morphology of the composite material under different laser scanning speeds, and Figure 4 shows the changes in the surface roughness of the composite material under different laser scanning speeds.

As shown in Figure 3, the scanning speed has a significant impact on the surface morphology of the TC4-B_4_C composite material. When the scanning speed is 400 mm/s, there are many pores and irregular undulations and much molten accumulation on the sample surface, and the overall surface is relatively rough. This is because at a low scanning speed, the laser stays in the same area for a longer time, resulting in significant heat accumulation. The material’s surface layer undergoes excessive melting and unstable recrystallization, leading to an increase in surface defects. After the scanning speed is increased to 500–600 mm/s, the surface roughness and accumulation decrease, and the pores and large-scale undulations gradually weaken, demonstrating that appropriately increasing the scanning speed can reduce excessive heat input and stabilize the molten pool state.

When the scanning speed increased to 700 mm/s, the surface morphology of the sample was further improved, with a significant reduction in surface undulations. The overall continuity and uniformity were good, demonstrating that at this point, the laser action time and heat input had reached a relatively reasonable match. The surface material could undergo sufficient and stable remelting flow, thereby achieving a better leveling effect. However, when the scanning speed continued to increase to 800 mm/s, although the surface was relatively uniform overall, there were still fine undulations and areas that were not fully leveled. This indicates that an excessively high scanning speed would shorten the laser–material interaction time, resulting in insufficient remelting depth and melt flow capacity on the surface, and consequently, the polishing effect would decline.

As shown in Figure 4, the change in roughness is basically consistent with the evolution of surface morphology. As the scanning speed increased from 400 mm/s to 700 mm/s, Sa decreased from 27.36 μm to 4.13 μm, and Sq decreased from 34.59 μm to 5.35 μm, revealing that appropriately increasing the scanning speed can significantly reduce the average surface undulation. At the same time, Sz decreased from 200.83 μm to approximately 51.33 μm, indicating that the maximum peak–valley difference of the surface has been effectively controlled. When the scanning speed continued to increase to 800 mm/s, Sa, Sq, and Sz returned to 8.84 μm, 14.10 μm, and 109.32 μm, respectively, revealing that at an excessively high scanning speed, the remelting effect is insufficient, and the local peak–valley difference increases again.

In conclusion, the scanning speed affects the polishing effect by altering the duration of the laser’s action on a unit area and the degree of heat accumulation. When the scanning speed is too low, there is excessive heat input, which can lead to melting accumulation, ablation, and fluctuations in re-solidification; when the scanning speed is too high, the heat action time is insufficient, making it difficult to fully eliminate the peaks and valleys on the original surface. Based on the surface morphology and roughness results, the samples at a scanning speed of 700 mm/s have the lowest Sa and Sq values and better surface uniformity. This indicates that the heat input and the stability of the molten pool are better matched at this speed, making it suitable as the reference scanning speed for subsequent experiments on scanning intervals.

(3) Influence of scanning interval

**Figure 5:**
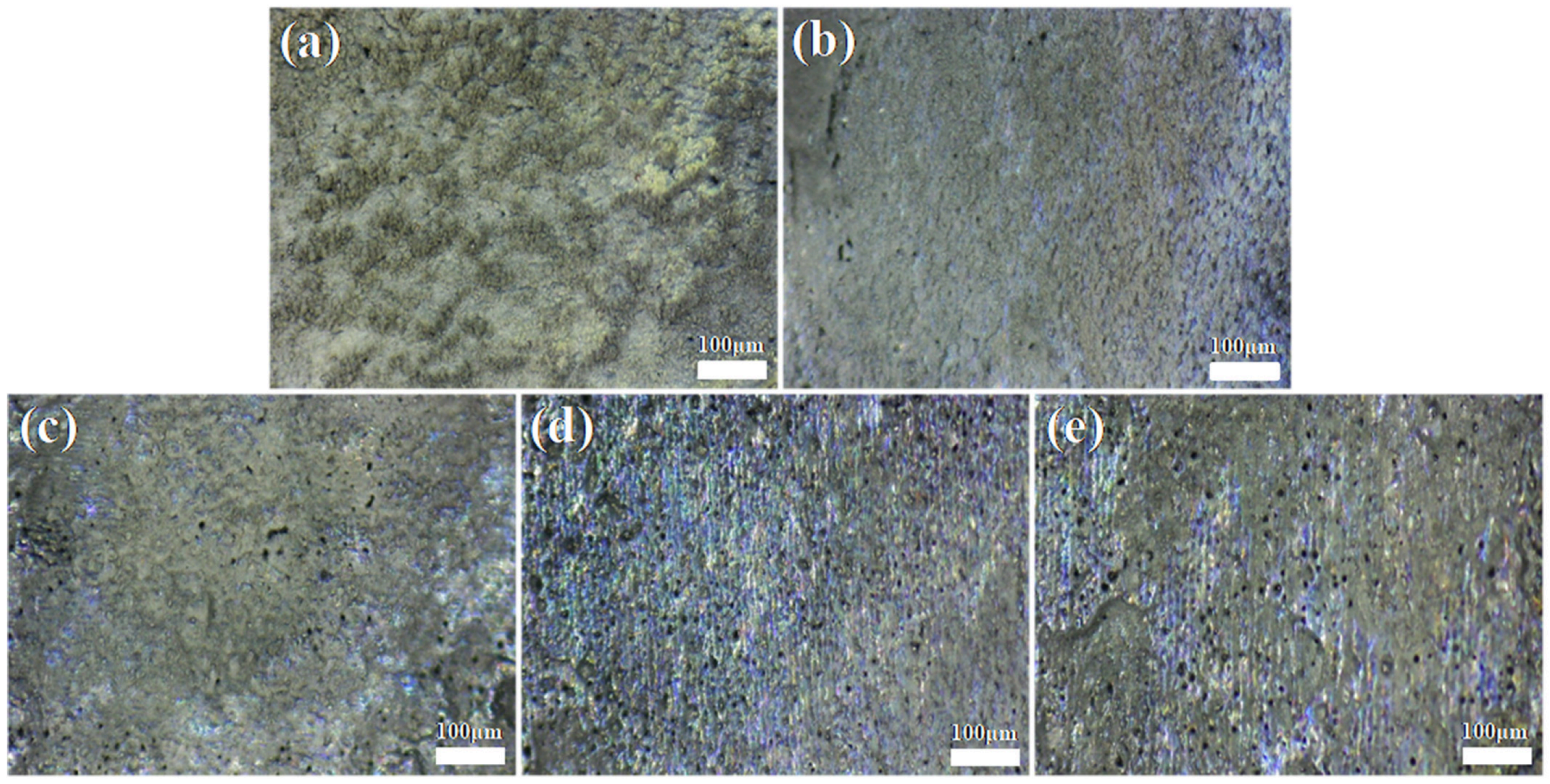
Changes in the surface morphology of the composite material under different scanning spacings (scale bar = 100 μm): (**a**) 0.01 mm; (**b**) 0.02 mm; (**c**) 0.03 mm; (**d**) 0.04 mm; (**e**) 0.05 mm.

**Figure 6:**
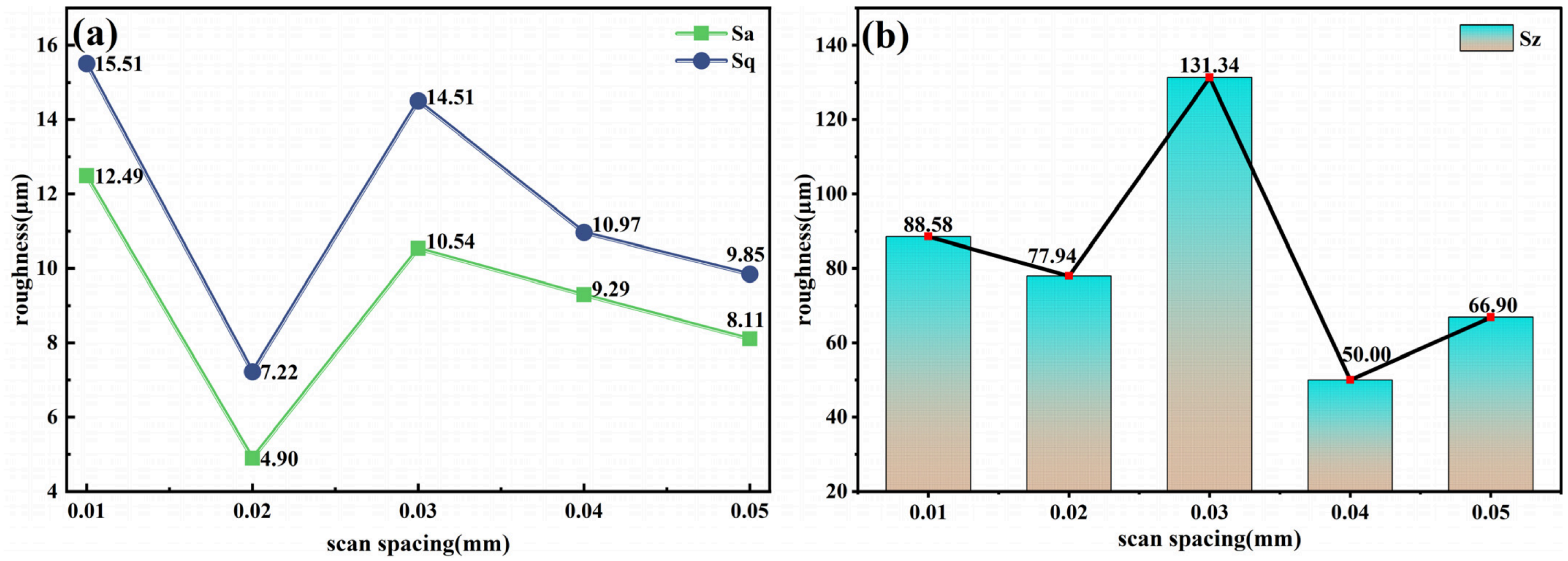
Changes in surface roughness of composite materials under different scanning intervals: (**a**) roughness Sa, Sq; (**b**) maximum height Sz.

Figure 5 shows the changes in the surface morphology of the composite material under different laser scanning intervals, and Figure 6 shows the changes in the surface roughness of the composite material under different laser scanning intervals.

As shown in Figure 5, the scanning interval has a significant impact on the surface morphology of the TC4-B_4_C composite material. When the scanning interval is 0.01 mm, there are many molten deposits and irregular undulations on the sample surface, and the overall uniformity is poor. This is because the scanning interval is too small, and the overlapping degree between adjacent scanning paths is too high. The material surface is repeatedly irradiated by the laser, resulting in cumulative local heat input and repeated remelting, thereby exacerbating the re-solidification undulations. After increasing the scanning interval to 0.02 mm, the surface morphology significantly improves, with fewer pores and rough deposits, and the overall continuity is enhanced. This indicates that at this time, the scanning paths are reasonably connected, which can ensure sufficient remelting of the surface and avoid excessive heat accumulation. When the scanning interval continues to increase to 0.03 mm, the sample surface again shows more obvious pores, grooves, and uneven undulations, revealing that the connection between adjacent scanning paths is insufficient, and some areas are not effectively remelted, resulting in a decrease in surface continuity. Upon further increasing it to 0.04–0.05 mm, the surface morphology slightly improves compared to 0.03 mm but still shows local undulations and discontinuous areas, revealing that an excessively large scanning interval will weaken the coverage effect between laser trajectories, resulting in a risk of insufficient connection in the polished area.

As shown in Figure 6, the change in roughness is basically consistent with the evolution of surface morphology. When the scanning interval increased from 0.01 mm to 0.02 mm, Sa decreased from 12.49 μm to 4.90 μm, and Sq decreased from 15.51 μm to 7.22 μm. This indicates that appropriately increasing the scanning interval can effectively reduce repeated remelting and thermal accumulation, significantly reducing the average surface undulation. When the scanning interval was 0.02 mm, both Sa and Sq reached their minimum values, which implies that this condition provided the best surface flatness effect. When the scanning interval was increased to 0.03 mm, Sa and Sq rose to 10.54 μm and 14.51 μm, respectively, and Sz reached 131.34 μm. This shows that insufficient overlap would significantly increase the local peak–valley difference. When the scanning interval was 0.04 mm, Sz dropped to 50.00 μm, but Sa remained at 9.29 μm, which implies that the local extreme peaks and valleys decreased, but the overall average undulation still did not receive sufficient improvement.

In conclusion, the scanning interval mainly affects the polishing effect by altering the degree of overlap between adjacent scanning paths. When the scanning interval is too small, repeated irradiation and thermal input accumulation can easily lead to repeated melting and re-solidification of the surface; when the scanning interval is too large, the adjacent scanning paths do not cover the surface adequately, which can easily result in unpolished areas and groove-like defects. Based on the surface morphology and roughness results, the samples have the lowest values of Sa and Sq under a scanning interval of 0.02 mm, with good surface continuity and uniformity, which implies that the thermal input distribution at this scanning interval matches the scanning path overlap state more optimally. The results of this single-factor experiment provide a basis for the selection of factor levels in subsequent orthogonal experiments and the optimization of process parameters.

### 3.2. Orthogonal Experiment Optimization and Parameter Impact Analysis

The results of the single-factor experiments indicated that the laser power, scanning speed, and scanning interval all affected the surface polishing quality of the TC4-B_4_C composite material. However, nanosecond laser polishing was not the result of the independent action of a single parameter; rather, it was the result of the combined effect of the heat input intensity, laser action time, and the overlapping state of the scanning paths. Therefore, in order to obtain a better process combination under the condition of multi-parameter coupling, this paper used Sa as the evaluation index and conducted an optimization analysis of the laser power, scanning speed, and scanning interval using the L9(3^3^) orthogonal test. The orthogonal test design and results are shown in Table 5.

As shown in Table 5, there are significant differences in the surface Sa values of the samples under different parameter combinations, which implies that there is a certain coupling effect among the laser power, scanning speed, and scanning interval. The Sa values of the nine orthogonal test samples were all lower than the initial roughness of the unpolished samples (5.98 μm), suggesting that the selected parameter range has an overall polishing effect. Among them, the sample in group A3 had the smallest Sa value of 2.100 μm, with the corresponding parameters being a laser power of 30 W, a scanning speed of 750 mm/s, and a scanning interval of 0.025 mm. Compared with the unpolished samples, the roughness decreased by 64.9%, and the surface leveling effect was the most obvious. The sample in group A7 had the largest Sa value of 5.374 μm, indicating that under the combination of higher power and lower scanning speed, the heat input was too high, and the polishing effect was relatively limited.

To further compare the influence degree of each factor on Sa, a range analysis was conducted on the results of the orthogonal experiment. The results are shown in Table 6. Here, the k value represents the average value of Sa under different levels of each factor, and the R value represents the range. The larger the R value, the more significant the influence of that factor on the surface roughness.

From Table 6, it can be seen that the range values of laser power, scanning speed, and scanning interval decrease successively, which implies that the influence of these three factors on surface roughness Sa is highest for laser power, then scanning speed, and lowest for scanning interval. According to the principle that a smaller k value corresponds to the preferred level, the preferred level of laser power is A1, the preferred level of scanning speed is B3, and the preferred level of scanning interval is C3. Therefore, the optimal parameter combination obtained from the orthogonal experiment is A1B3C3, that is, a laser power of 30 W, a scanning speed of 750 mm/s, and a scanning interval of 0.025 mm. This combination is consistent with the experimental result of the orthogonal experiment A3 group and corresponds to the minimum Sa value, indicating that the range analysis results have a good consistency with the experimental test results.

From the perspective of the mechanism of action, the laser power mainly determines the intensity of heat input per unit area, directly influencing the melting and recrystallization state of the surface layer; the scanning speed affects the accumulation of heat and the stability of the molten pool by changing the laser action time; the scanning interval regulates the overlapping state of adjacent scanning paths and affects the continuity of the remelting layer. Since the range of variation in the scanning interval in this experiment is relatively small, its influence is weaker than that of the laser power and the scanning speed. Combining the results of the single-factor experiments, it can be known that the order of influence obtained from the range analysis is consistent with the variation pattern of surface morphology and roughness, indicating the rationality of the optimization results. In conclusion, 30 W, 750 mm/s, and 0.025 mm are determined as the optimal parameters for nanosecond laser polishing, and they are used for subsequent surface quality research and analysis.

The combination of single-factor and orthogonal experiments effectively defines a practical process window for the nanosecond laser polishing of the TC4-B_4_C composite (specifically around 30–40 W, 650–750 mm/s, and 0.015–0.025 mm). Within this window, the laser thermal input perfectly matches the melting and flowing behavior of the multiphase material. Furthermore, the highly uniform surface morphology and consistent remelted features observed across the entire treated area (as will be shown in the subsequent SEM analysis) serve as a strong indication of processing repeatability. This macroscopic and microscopic uniformity demonstrates that the selected optimal parameters can provide a stable melt pool state, effectively mitigating the risk of localized random defects and ensuring reproducible surface finishing.

### 3.3. Surface Microscopic Morphology and Elemental Characteristics Under Optimal Parameters

**Figure 7:**
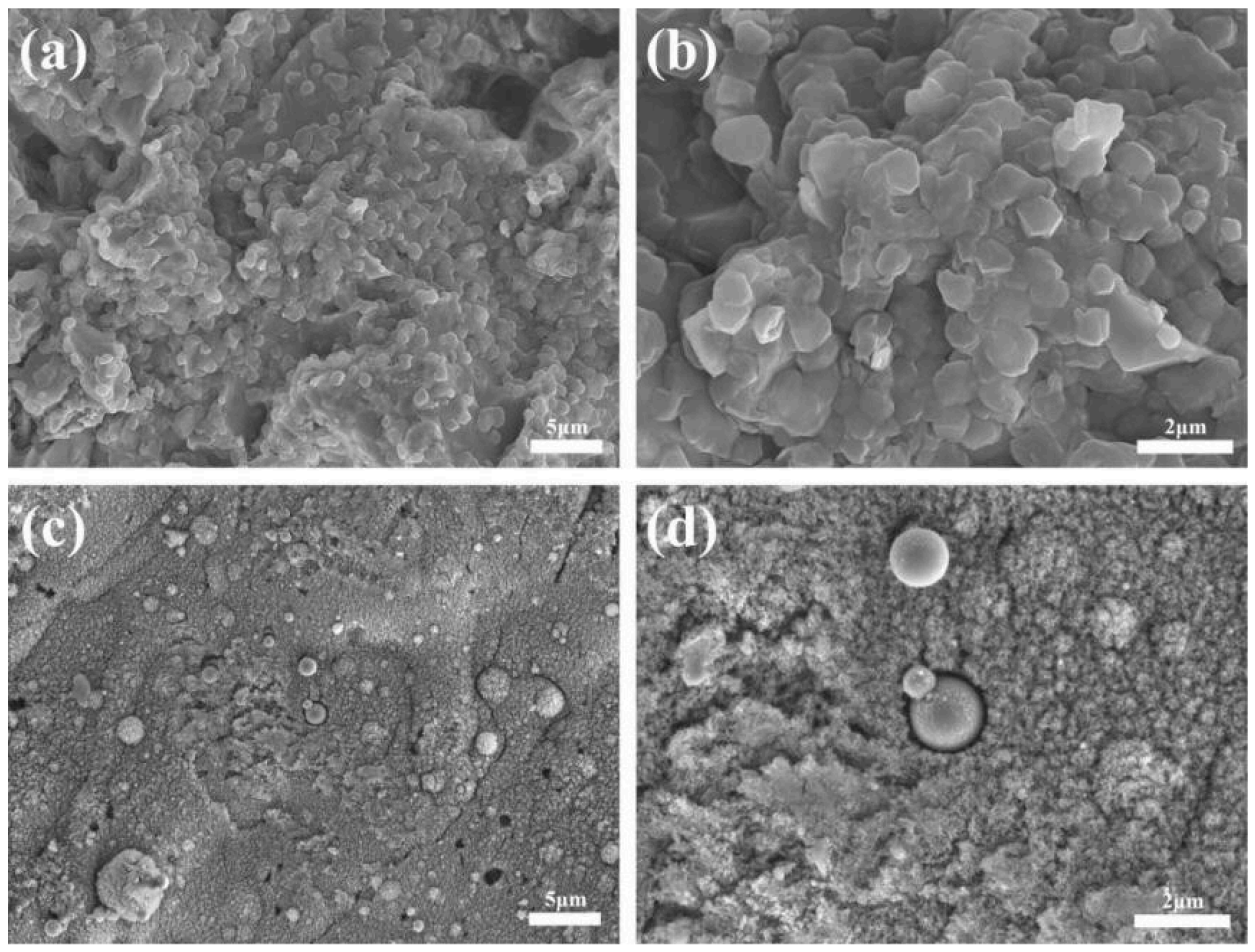
SEM morphology comparison between unpolished samples and samples polished with the optimal parameters applied: (**a**,**b**): unpolished samples; (**c**,**d**): samples polished with the optimal parameters applied.

To further verify the effect of the optimal parameters obtained from the orthogonal experiment on improving the surface quality, SEM and EDS comparative analyses were conducted on the unpolished samples and the samples with the optimal parameters applied. Among them, the optimal parameters were a laser power of 30 W, a scanning speed of 750 mm/s, and a scanning interval of 0.025 mm. The aforementioned results indicated that the sample’s Sa was the lowest under these parameters, but it was difficult to comprehensively reveal the microscopic reasons for the improvement in surface quality solely based on the roughness values. Therefore, it is necessary to conduct further analysis from the perspectives of surface morphology and elemental characteristics. Figure 7 shows the SEM morphology comparison between the unpolished sample and the sample polished with the optimal parameters applied. (a) and (c) are the morphologies at 3000× magnification, and (b) and (d) are the morphologies at 10,000× magnification.

As shown in Figure 7, the unpolished sample surface exhibits obvious rough accumulation, pores, and irregular undulations, with local areas showing loose and discontinuous characteristics. This is related to the initial defects and particle bonding state of the surface of the powder-metallurgy-prepared samples. After nanosecond laser polishing, the surface morphology of the sample has significantly improved. The original rough protrusions and pore defects have been weakened, and a relatively continuous remelting area has formed on the surface layer, with the overall uniformity enhanced. The reason lies in the fact that the laser thermal input under the optimal parameters is relatively moderate. The surface micro-convex peaks are preferentially melted, and the molten material flows towards the low-lying areas under the surface tension and fills the local pores. Subsequently, it rapidly solidifies to form a relatively dense remelting layer, thereby reducing the peak–valley difference on the surface and improving the surface flatness. Additionally, a few individual spherical particles can be observed on the polished surface, particularly in Figure 7c,d. These particles are most likely spatters generated during the transient laser melting process. The rapid localized energy input from the nanosecond laser can cause minor vaporization or gas expansion within the melt pool, occasionally expelling tiny molten droplets that rapidly cool and solidify into spherical spatters upon landing on the surrounding surface.

**Figure 8:**
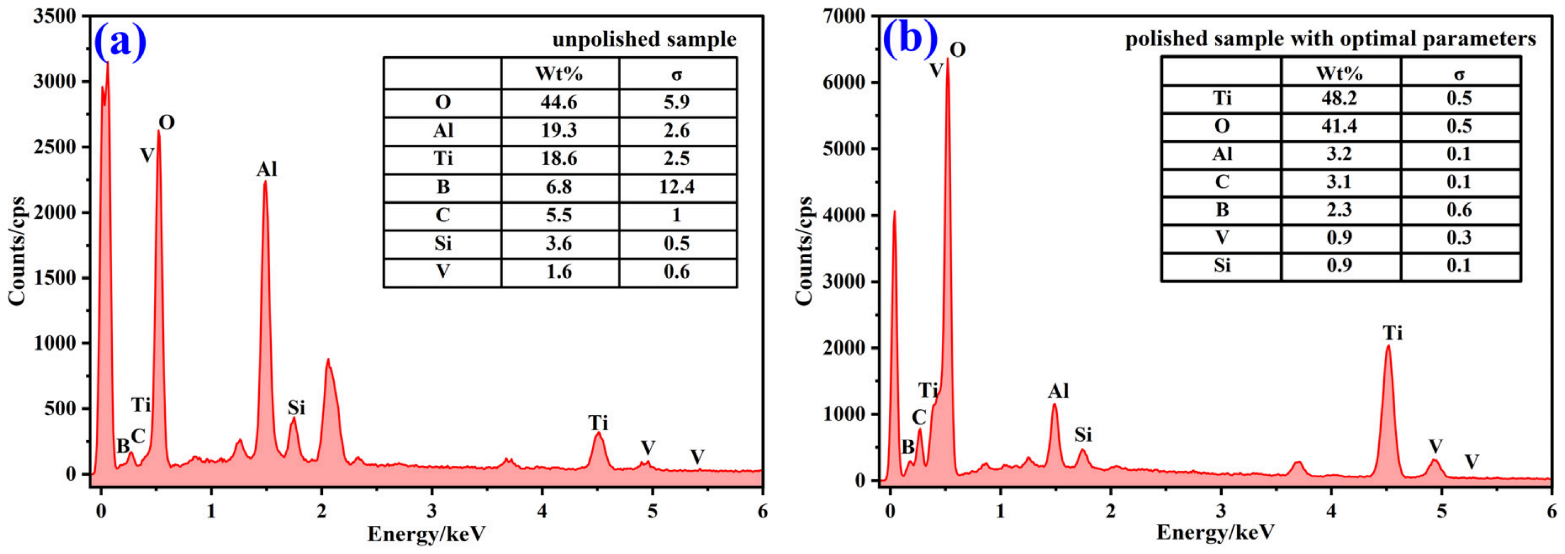
EDS element comparison of unpolished samples and samples polished with the optimal parameters applied: (**a**) unpolished sample; (**b**) sample polished with the optimal parameters applied.

Figure 8 shows the EDS element comparison diagram of the unpolished sample and the polished sample with the optimal parameters applied. The EDS analysis results indicate that the main element types on the surface of the samples before and after polishing have not undergone fundamental changes, still mainly consisting of elements such as Ti and O. This suggests that the nanosecond laser polishing did not introduce a new element system. Its effect mainly manifested as surface remelting, tissue reconstruction, and adjustment of the oxidation state. Compared with the unpolished sample, the relative content of the Ti element in the optimal-parameter-polished sample increased from 18.6 wt% to 48.2 wt%, while the content of the O element slightly decreased from 44.6 wt% to 41.4 wt%, and the content of the Al element decreased from 19.3 wt% to 3.2 wt%. The element distribution on the surface of the optimal-parameter sample is more uniform, indicating that laser polishing weakened the original loose coating and local component fluctuations, making the surface remelting layer more stable. Combined with the SEM morphology, it can be seen that the improvement in surface quality under the optimal parameters mainly comes from rapid remelting under moderate heat input conditions, the filling of the molten material, and the rapid solidification process, rather than a fundamental change in the material composition system.

In conclusion, the parameter combination of 30 W, 750 mm/s, and 0.025 mm can promote the reconstruction of the surface structure and form a relatively continuous and dense remelted layer without significantly changing the types of main elements on the surface layer, thereby effectively reducing surface pores, rough accumulation, and irregular undulations. This result further demonstrates the rationality of orthogonal optimization parameters from the perspectives of microscopic morphology and elemental characteristics; it also provides an organizational basis for subsequent microhardness and phase analysis.

**Figure 9:**
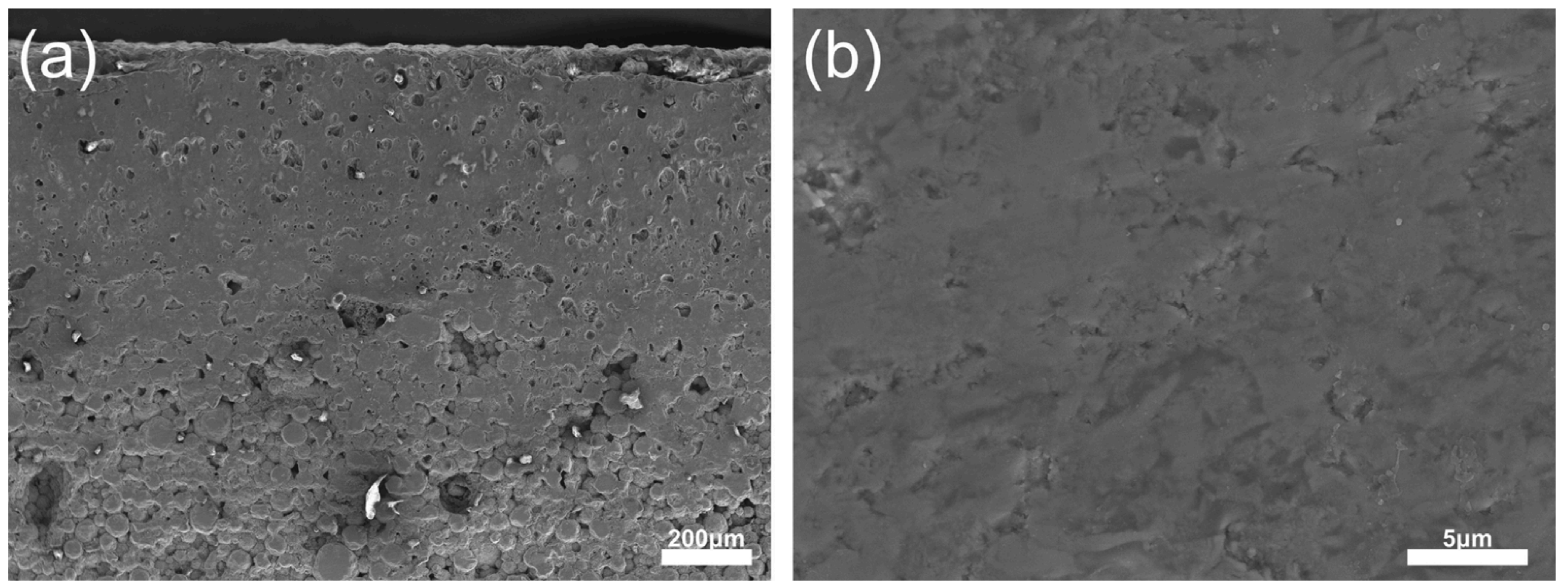
Cross-sectional SEM morphology of the A3 sample treated with the optimal parameters of 30 W, 750 mm/s, and 0.025 mm applied: (**a**) low-magnification cross-sectional image with thickness measurements (scale bar = 200 μm); (**b**) high-magnification morphology of the laser-modified remelted layer (scale bar = 5 μm).

To directly evaluate the structural integrity of the polished surface, cross-sectional SEM observation was performed on the sample treated with the optimal parameters of 30 W, 750 mm/s, and 0.025 mm applied. As shown in Figure 9, a continuous laser-modified remelted layer was observed near the top surface of the sample. The thicknesses measured at three different positions were 865.78 μm, 847.91 μm, and 866.97 μm giving an average thickness of 860.2 ± 10.7 μm. No obvious through-thickness cracks or interfacial delamination were observed in the inspected regions. The interface between the laser-modified layer and the underlying matrix appeared to be continuous, indicating that the optimized nanosecond laser polishing parameters produced acceptable structural integrity. Meanwhile, several isolated pores and particle-related cavities could still be observed in the cross-section, which is consistent with the inherent porous and heterogeneous characteristics of the powder-metallurgy-derived TC4-B_4_C composite.

### 3.4. Surface Hardness and Phase Analysis

To further evaluate the influence of nanosecond laser polishing on the surface properties and phase state of TC4-B_4_C composite materials, a comparative analysis of microhardness and XRD was conducted between the unpolished samples and the samples with the optimal parameters applied. Among them, the optimal parameters were a laser power of 30 W, a scanning speed of 750 mm/s, and a scanning interval of 0.025 mm.

The results of microhardness testing show that the surface hardness of the unpolished sample is 704 HV, and the surface hardness of the sample polished with the optimal parameters applied increases to 829.3 HV, with an increase of 17.8%. This indicates that nanosecond laser polishing not only reduces the surface roughness but also has a certain strengthening effect on the material’s surface layer. The reason for this is that the moderate laser heat input causes the material surface layer to undergo rapid remelting and recrystallization, weakening the original loose areas, pores, and rough accumulations, and forming a relatively continuous and dense remelting layer on the surface; at the same time, the rapid solidification process may promote local microstructure refinement, thereby enhancing the surface layer’s ability to resist plastic deformation.

It should be noted that the maximum microhardness value in the orthogonal experiment does not exactly correspond to the sample with the lowest roughness. The A5 sample has the highest hardness of 859.4 HV, while the A3 sample, which has the best roughness, has a hardness of 829.3 HV, still remaining at a relatively high level. Since this paper takes the surface roughness Sa as the main optimization indicator, the A3 sample achieves the lowest Sa while maintaining a relatively high microhardness. This indicates that the parameter combination has achieved a better balance between surface smoothing and surface strengthening, giving this sample superior comprehensive surface quality.

**Figure 10:**
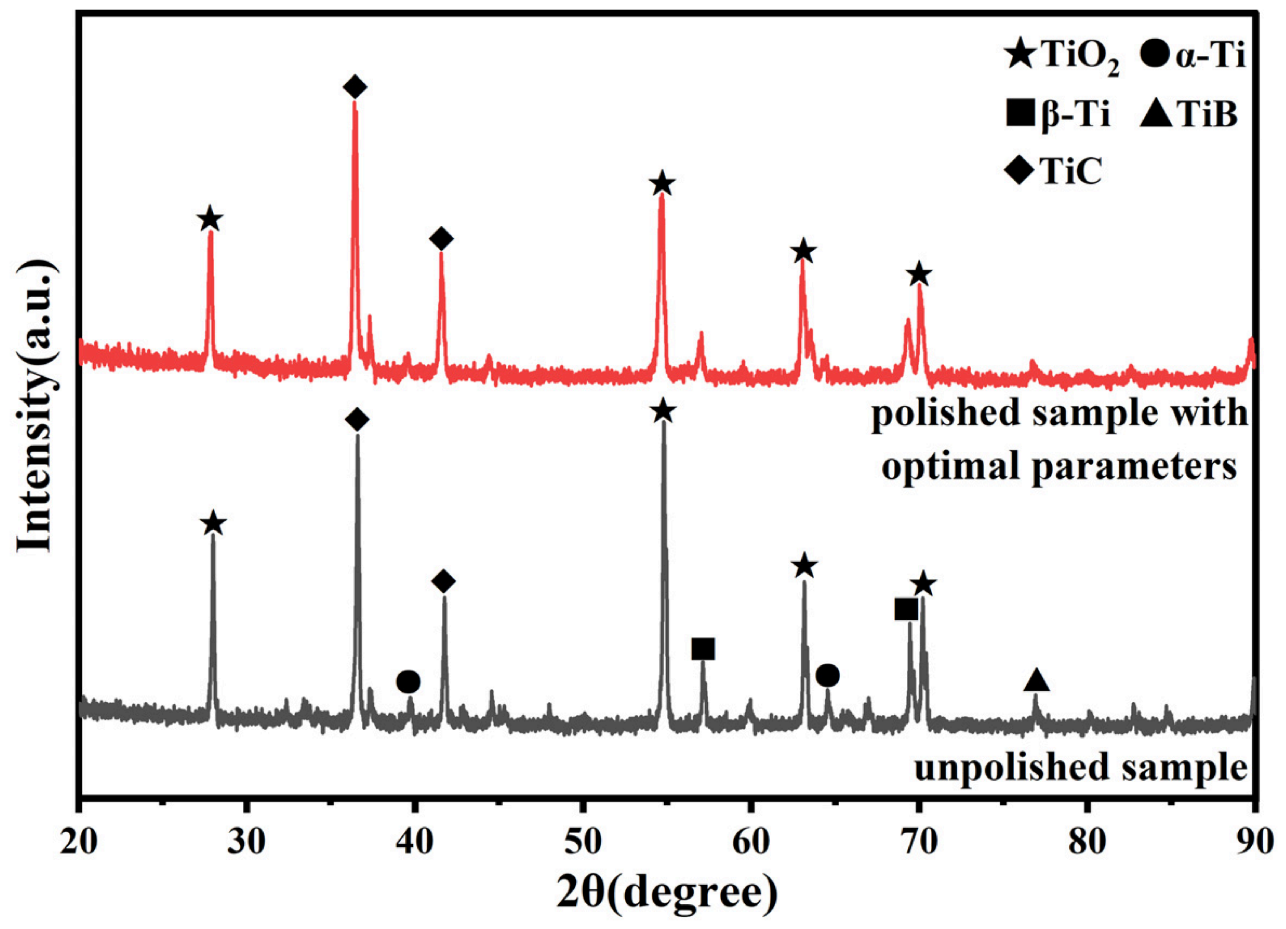
Comparison of XRD spectra between the unpolished sample and the polished sample with the optimal parameters applied.

Figure 10 shows a comparison diagram of the XRD spectra of the unpolished sample and the sample polished with the optimal parameters applied. The XRD analysis results indicate that the main diffraction peak types of the unpolished sample and the sample polished with the optimal parameters applied are basically the same. The surface is still dominated by TiO_2_-, TiC- and Ti-based related phases, and no new main phase is observed. After polishing, the intensity of some TiO_2_-related peaks changes, suggesting that the surface has undergone a rapid thermal cycling and local oxidation process under the action of the nanosecond laser, and the relative content and oxidation state of the phases have been adjusted. At the same time, the characteristics of the TiC and other reinforcing phases are still retained, suggesting that the laser polishing has not disrupted the original main phase system of the composite material.

Based on the comprehensive hardness and XRD results, it can be concluded that under the optimal parameters, the nanosecond laser polishing of the TC4-B_4_C composite material mainly exhibits the effects of surface remelting, microstructure reconstruction, and adjustment of the oxidation state, rather than the formation of a new main phase. This process not only improves the surface flatness but also enhances the microhardness of the surface layer, further verifying the effectiveness of the parameter combination of 30 W, 750 mm/s, and 0.025 mm in improving surface quality and enhancing the performance of the surface layer.

### 3.5. Discussion on Novelty and Process Specificity

Compared to the nanosecond laser polishing of homogeneous Ti6Al4V alloys or standard particle-reinforced composites, the TC4-B_4_C system presents unique challenges due to its multiphase nature. The differences in thermal conductivity and melting behavior between the titanium matrix and the B4C reinforcing phase can lead to localized thermal gradients and viscosity variations within the transient melt pool. Table 7 summarizes a comparison between the optimal polishing results of this study and representative laser polishing works on titanium-based materials. While traditional laser polishing of additive manufactured Ti6Al4V typically achieves significant roughness reduction, the presence of hard ceramic phases often hinders melt flow, making it difficult to achieve a smooth surface without inducing thermal cracks or severe ablation.

In this study, by optimizing the process window (specifically within 30–40 W, 650–750 mm/s, and 0.015–0.025 mm), the nanosecond laser not only accommodated the thermophysical mismatch of the TC4-B_4_C system but also achieved a 64.9% reduction in Sa alongside a 17.8% increase in microhardness. This demonstrates that within the identified practical process window, the transient thermal input is perfectly balanced to allow sufficient melting of the multiphase surface without triggering deleterious phase agglomeration or deep ablation.

The significant improvements in both surface topography and microhardness achieved by the optimized nanosecond laser polishing are theoretically expected to translate directly into enhanced service performance for the TC4-B_4_C composite, particularly in terms of wear resistance and fatigue life. From a tribological perspective, the substantial reduction in surface arithmetic mean height Sa (down to 2.1 μm) minimizes the presence of sharp surface asperities, which typically act as abrasive sites and increase friction during dry sliding. Concurrently, the 17.8% increase in surface microhardness (up to 829.3 HV) effectively enhances the load-bearing capacity and the resistance to plastic deformation of the remelted layer, which is anticipated to synergistically reduce the wear rate. Furthermore, regarding dynamic loading scenarios, it is widely recognized that surface defects (such as open pores, irregular valleys, and coarse accumulations) serve as primary stress concentration sites and fatigue crack initiation points in titanium-based materials. The dense, continuous, and defect-healed remelted layer formed after laser polishing can effectively reduce the primary surface fatigue initiation sites, strongly implying an extended fatigue life for the TC4-B_4_C composites in actual engineering applications. Recent studies on laser-processed titanium alloy surfaces have suggested that in situ formed hard ceramic phases, such as TiC and Ti(C,N), can act as load-bearing reinforcement phases, suppress micro-cutting damage during sliding wear, and thereby improve wear resistance [19,20]. However, since the present work did not directly evaluate wear behavior, the potential tribological benefit of the optimized laser-polished surface still requires further verification through future wear tests.

## 4. Conclusions

This paper takes a 90 wt% TC4 + 5 wt% B_4_C + 5 wt% SiO_2_ composite material as the research object and conducts nanosecond laser polishing experiments. It analyzes the influence laws of process parameters on surface quality and surface properties and obtains the following conclusions:

(1) The laser power, scanning speed, and scanning interval all affect the polishing effect of the TC4-B_4_C composite material surface. Among them, the laser power mainly affects the heat input intensity, the scanning speed mainly regulates the degree of heat accumulation, and the scanning interval mainly determines the overlapping state of the scanning path. Insufficient heat input will lead to insufficient remelting of the surface layer, and excessive heat input is likely to cause over-melting, ablation, and fluctuations in re-solidification, all of which are not conducive to improving the surface quality.
(2) The orthogonal test results show that the order of influence of the three factors on the surface roughness Sa is laser power, then scanning speed, then scanning interval. The optimal process parameters are a laser power of 30 W, a scanning speed of 750 mm/s, and a scanning interval of 0.025 mm. Under these parameters, the surface Sa of the sample drops to 2.1 μm, which is 64.9% lower than that of the unpolished sample (5.98 μm).
(3) After polishing with the optimal parameters, the surface roughness accumulation, pores, and irregular fluctuations are significantly reduced, forming a relatively continuous and dense remelt layer, and the surface uniformity is improved. EDS analysis indicates that the main element types before and after polishing have not undergone fundamental changes, and the laser effect mainly manifests as the re-structuring of the surface layer and the adjustment of the oxidation state.
(4) Under the optimal parameters, the microhardness of the sample surface increases from 704 HV to 829.3 HV, an increase of 17.8%. XRD results show that the surface layer after polishing is mainly composed of TiO_2_-, TiC-, and Ti-based related phases, and no new main phase is formed. Comprehensive analysis suggests that nanosecond laser polishing mainly forms a dense remelt layer through rapid remelting of the surface layer, filling of the molten body, and rapid solidification, thereby achieving surface leveling and improvements in surface properties.

## Figures and Tables

**Table 1:** Specific parameters of the nanosecond laser.

Parameter Name	Parameter Scale
Wavelength	1064 nm
Maximum power	60 W
Scanning speed	1–15,000 mm/s
Frequency	20~200 kHz
Cooling method	Air cooling
Scanning range	150 mm × 150 mm
Focal length	319 mm
Pulse width	10 ns
Spot radius	50 μm

**Table 2:** Parameters of nanosecond laser polishing fixation process.

Parameters	Numerical Values or Conditions
Laser repetition frequency	30 kHz
Pulse width	10 ns
Scanning times	1 time
Incidence mode	Vertical incidence
Polishing area	5 mm × 5 mm
Scanning method	Bow-shaped scanning

**Table 3:** Experimental process parameters of single-factor experiment.

Sample Number	Laser Power/W	Scanning Speed/(mm/s)	Scanning Spacing/mm
1	10	500	0.02
2	20	500	0.02
3	30	500	0.02
4	40	500	0.02
5	50	500	0.02
6	40	400	0.02
7	40	500	0.02
8	40	600	0.02
9	40	700	0.02
10	40	800	0.02
11	40	700	0.01
12	40	700	0.02
13	40	700	0.03
14	40	700	0.04
15	40	700	0.05

**Table 4:** Orthogonal experiment factor level table.

Level	Laser Power/W	Scanning Speed/(mm/s)	Scanning Spacing/mm
1	30	650	0.015
2	35	700	0.02
3	40	750	0.025

**Table 5:** Orthogonal experimental design and Sa results.

Experiment Number	Laser Power/W	Scanning Speed/(mm/s)	Scanning Spacing/mm	Sa/μm
A1	30	650	0.015	3.883
A2	30	700	0.020	3.253
A3	30	750	0.025	2.100
A4	35	650	0.020	4.811
A5	35	700	0.025	4.339
A6	35	750	0.015	2.897
A7	40	650	0.025	5.374
A8	40	700	0.015	5.146
A9	40	750	0.020	4.758
A10		Unpolished		5.980

**Table 6:** Orthogonal experiment analysis of variance.

Item	Level	A Laser Power	B Scanning Speed	C Scanning Interval
K value	1	9.236	14.068	11.926
	2	12.047	12.738	12.822
	3	15.278	9.755	11.813
K value	1	3.079	4.689	3.975
	2	4.016	4.246	4.274
	3	5.093	3.252	3.938
Range R		2.014	1.437	0.336

**Table 7:** Comparison between the present nanosecond laser polishing results and representative laser-based surface modification studies on titanium-based materials.

Material System	Method	Process Condition	Representative Result	Main Feature
Ti6Al4V (SLM)	Laser polishing	Optimized LP-2 condition	~79.4% roughness reduction [7]	Effective surface smoothing of additively manufactured Ti alloy
Ti6Al4V (LPBF)	Laser polishing	Optimized CO_2_ laser polishing condition	~80.0% roughness reduction [17]	Improved surface quality of LPBF Ti-6Al-4V
Ti(C,N)-reinforced coating on TC4	Laser cladding	4# coating with high TiN/Ti/C addition	Wear volume reduced to 0.145 times that of TC4 substrate [19]	TiC/Ti(C,N) hard phases suppress micro-cutting
TC4-B_4_C composite	Nanosecond laser polishing	30 W, 750 mm/s, hatch spacing of 0.025 mm	Sa reduced by 64.9%; hardness increased to 829.3 HV	Optimized polishing of PM multiphase Ti-based composite

## Data Availability

The original contributions presented in the study are included in the article; further inquiries can be directed to the corresponding author.
